# The effect of shock waves on mineralization and regeneration of distraction zone in osteoporotic rabbits

**DOI:** 10.1080/07853890.2023.2192958

**Published:** 2023-03-30

**Authors:** Enes Özkan, Erman Şenel, Mehmet Cihan Bereket, Mehmet Emin Önger

**Affiliations:** a Department of Oral and Maxillofacial Surgery, Faculty of Dentistry, Istanbul Medeniyet University, Istanbul, Turkey; b Department of Oral and Maxillofacial Surgery, Modern Oral and Dental Health Center, Kocaeli, Turkey; c Department of Oral and Maxillofacial Surgery, Ondokuz Mayıs University, Faculty of Dentistry, Samsun, Turkey; d Department of Histology and Embryology, Ondokuz Mayıs University, Faculty of Medicine, Samsun, Turkey

**Keywords:** Distraction osteogenesis, dual-energy x-ray absorptiometry, extracorporeal shock wave therapy, osteoporosis, stereology

## Abstract

**Objective:**

Osteoporotic individuals suffer from various complications such as spontaneous bone fractures due to decreased bone strength and failure in bone healing as a result of decreased bone mineral density and deterioration of bone microstructure. In this study, the effects of Extracorporeal Shock Wave Therapy (ESWT) in a distraction osteogenesis model in osteoporotic rabbits were investigated to prevent these failures and improve bone microstructure.

**Material and Methods:**

A total of 28 female New Zealand rabbits underwent mandibular distraction osteogenesis and were divided into four groups: non-ovariectomized control (Cont), ovariectomized control (O-Cont), ovariectomized ESWT1 (O-ESWT1) and ovariectomized ESWT2 (O-ESWT2). ESWT was only applied to the ESWT2 group before the osteotomy, and to both the ESWT1 and ESWT2 groups after the osteotomy. Dual-energy x-ray absorptiometry was used to determine bone mineral density on both the 7th and 28th day of the consolidation. Stereological methods were used to identify new bone formation, connective tissue and neoangiogenesis volume.

**Results:**

According to the dual-energy x-ray absorptiometry examination both at the 7th and 28th day of the consolidation, lower bone mineral density was seen in the ESWT groups. However, the stereological examination showed that shock wave therapy significantly increased new bone formation both ESWT1 and ESWT2 compared with O-Cont, significantly increased neoangiogenesis in O-ESWT1 compared with O-Cont.

**Conclusions:**

The application of ESWT in these parameters after osteotomy was beneficial for bone regeneration in mandibular distraction in osteoporotics. However, ESWT has been shown to be ineffective in improving bone mineral density.

**KEY MESSAGES**
1. The osteoporotic model can be successfully established in rabbits and the subjects can tolerate the distraction procedures.
2. Stereology is a useful analysis method that can determine the volume of the new bone formation and neoangiogenesis.
3. Extracorporeal shock wave therapy has biostimulatory effects on bone tissue.

## Introduction

Osteoporosis is a metabolic bone disease characterized by a decreased bone mass and bone mineral density, and impaired bone microstructure. It is common, especially among postmenopausal women [1]. Due to these changes in bone structure, certain complications are observed, including susceptibility to bone fractures, delayed fracture healing, and loss of stabilization [2]. Decreased bone mineral density and increased cancellous bone spaces in the jaws of osteoporotic individuals are risk factors for early and late dental implant failure [3]. To prevent complications and reduce mortality and morbidity, standard treatments must be supported by inducing therapies for bone healing [4]. Although research on osteoporotic fractures has focused on preventing bone fractures and preserving the bone structure, little emphasis has been placed on bone healing [5].

Distraction osteogenesis (DO) is a generally accepted method for treating congenital and acquired deformities and inadequacies. It is based on gradually separating two bone fragments using an osteotomy to create new bone tissue [6,7]. In addition to being a long-drawn-out treatment, which is considered a disadvantage, there is a risk of stress-shielding osteopenia and osteoporosis in the bone due to the excessive load exerted by the distractors and screws [6]. There is a %38 possibility of recurrence of a fracture around the distractor or in the regeneration due to this osteoporotic condition [8]. Numerous physical or interventional methods have been analyzed to induce regeneration, including local application of growth factors, transplantation of osteoblast cell lines, local gene therapy, low-level laser therapy, and electrical and ultrasonic stimulation [6]. None of these approaches have yet been used in clinical practice. Therefore, it is critical to investigate effective non-invasive methods that might shorten the consolidation time and reduce stress-protective osteoporosis by inducing new bone formation in DO, even under poor osteogenic conditions.

The application of ESWT, which has proven to have a bio-stimulatory effect on osteogenesis, is a non-invasive treatment method currently used in treating many musculoskeletal diseases, including plantar fasciitis, calcific tendinitis of the shoulder, and delayed union or non-union. Shock waves used in the treatment are short, high-energy waves that can spread between tissues [9]. In the last ten years, ESWT has been proven to increase bone repair and regeneration by triggering the release of transcription factors, mediators, and growth factors [5,7,10]. Due to these positive effects, shock waves are thought to have promising results in the treatment of fracture healing, osteonecrosis, osteoarthritis, and osteoporosis [7,9]. Until now, studies of the effect of ESWT on osteoporotic bone tissue healing have been limited, and the focus of these has been to accelerate fracture healing [4,5].

The investigators hypothesized that Extracorporeal Shock Wave Therapy (ESWT) induce the bone healing and allow the formation of more calcified bone tissue in osteoporosis. The primary purpose of the study was to analyze the effects of ESWT on callus formation resulting from DO in a well-established osteoporotic rabbit model and the secondary purpose was to determine the appropriate application parameters of ESWT for the most optimal results. The specific aim of the study was to measure the parameters of interest in healthy and osteoporotic subjects and compare it between groups.

## Materials and methods

### Study design

This study received approval from the Ondokuz Mayıs University Animal Experiments Local Ethics Committee. All experiments complied with the National Research Council’s Guide for the Care and Use of Laboratory Animals. A total of 28 female New Zealand rabbits, aged 6-9 months and weighing an average of 2.75 kg were used in the study. The subjects were kept in separate cages with a 12-h day/night cycles, and were provided with standard food and water support throughout the study. The same DO protocol was applied to each subject with the same custom-made distractor. After the test animals were selected, they were randomly divided into four groups, with seven rabbits in each group:

**Cont:** Non-ovariectomized control

**O-Cont:** Ovariectomized control

**O-ESWT1:** Ovariectomized, ESWT applied after the osteotomy

**O-ESWT2:** Ovariectomized, ESWT applied before and after the osteotomy

### Osteoporotic model

**Figure 1.**
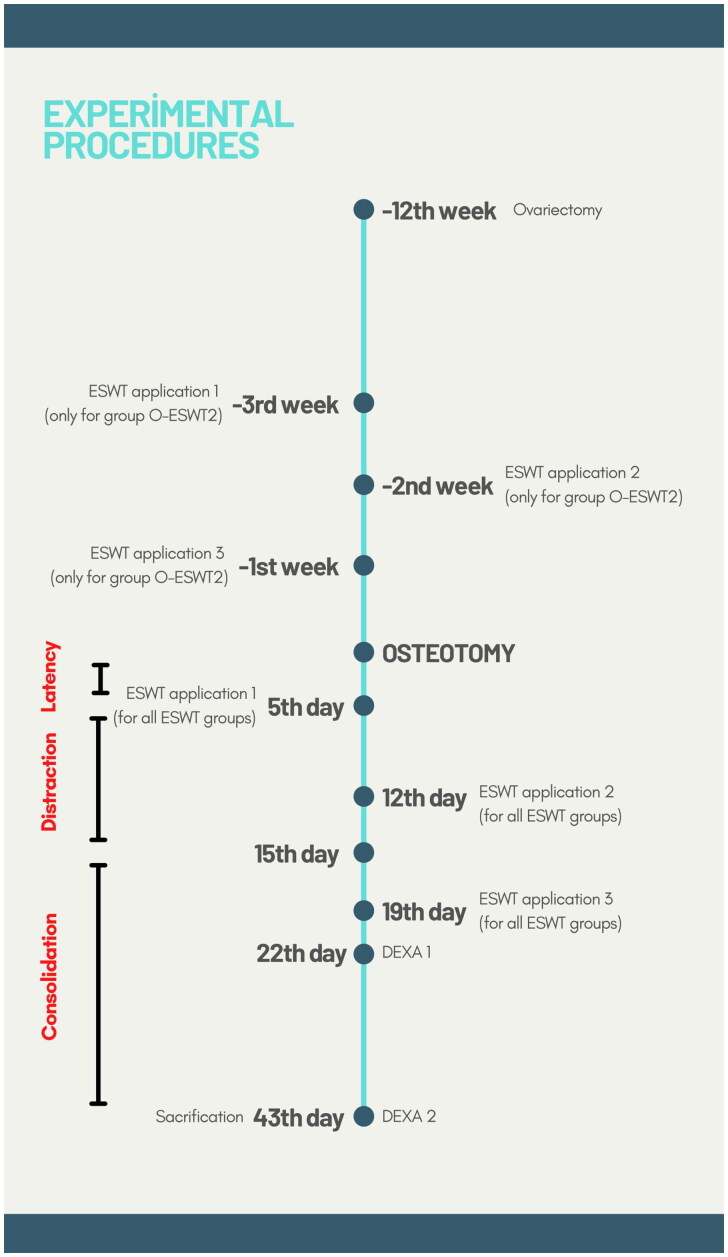
Timeline of experiment procedures.

The animals to be operated on were not fed the previous day. Each animal was administered 50 mg/kg ketamine HCL and 8 mg/kg xylazine HCL intramuscularly for general anaesthesia. Before the procedure, cefazolin sodium 50 mg/kg for prophylaxis and tramadol 1 mg/kg for analgesia were given by intramuscular injection. After shaving the abdomen of the anaesthetized animals, they were wiped with a povidone-iodine solution providing antisepsis in the surgical area. In the experimental groups which were to have an ovariectomy, the abdominal cavity was opened with a 4 cm laparotomy incision on the midline of the abdomen. After reaching the ovaries, the mesovarium and fallopian tube were ligated and the ovarian tissues on both sides were excised. In the subjects in the Cont group, the ovarian tissues were accessed after the abdominal cavity was opened similarly but were left *in situ* without being excised. All animals abdominal wall, subcutaneous, and skin tissues were sutured in layers and then closed. The procedure was concluded by applying antiseptic and antibiotic wound pomade, nitrofurazone 0.2%. After surgery, animals were placed in cages and intramuscular injections of cefazolin sodium 50 mg/kg and tramadol HCl 1 mg/kg were administered for three days in the postoperative period to prevent infection and provide analgesia. A period of 12 weeks were waited for the development of osteoporosis (Figure 1).

### ESWT device and ESWT application protocol before the osteotomy

**Figure 2.**
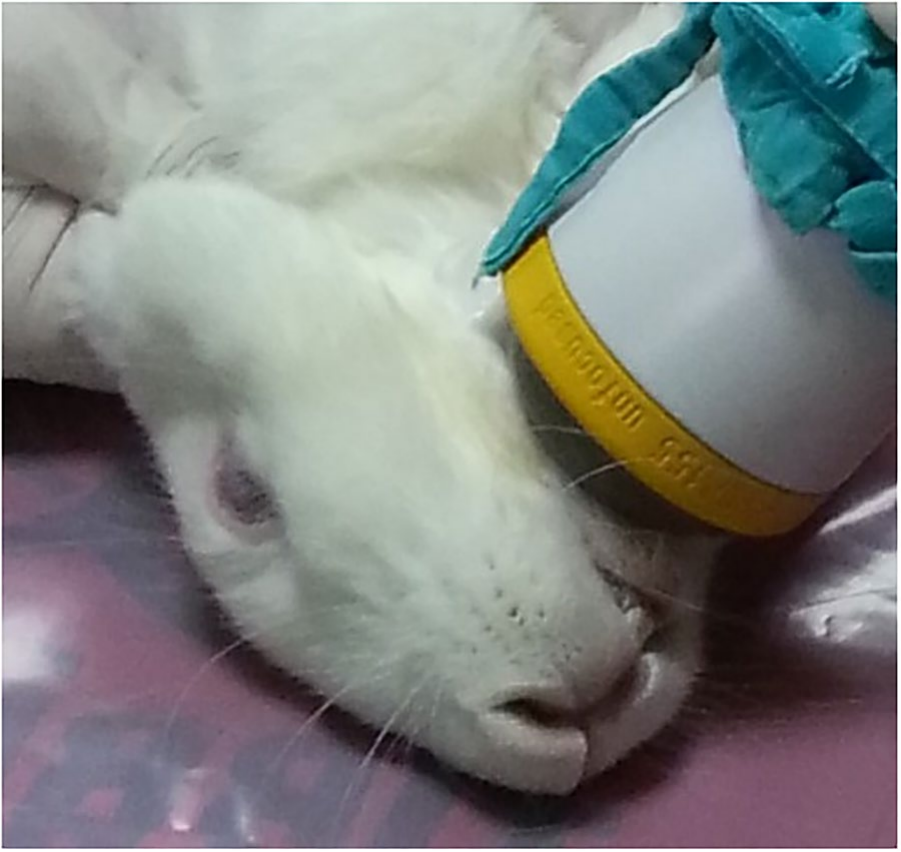
Unfocussed applicator of ESWT and shock wave application to distraction area.

The ESWT device with an unfocussed applicator were used in the study. Before applying ESWT, a lubricating gel was applied to the skin over the osteotomy site. The applicator was positioned perpendicular to the mandible in the submandibular region just below the osteotomy area (Figure 2). The rabbits in the O-ESWT2 group underwent ESWT at 500 impulses, 5 Hz, and 0.19 mJ/mm^2^ energy flow density parameters at the distraction site in the first, second and third weeks before the osteotomy.

### Osteotomy and distraction protocol

**Figure 3.**
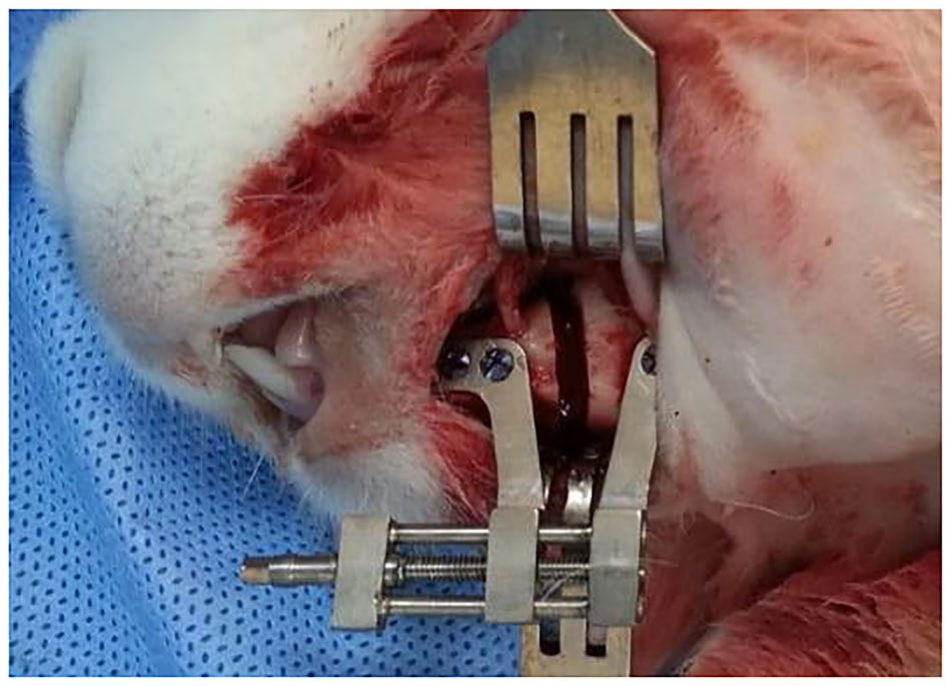
The custom-made titanium distractor and osteotomy line is posterior to the mental foramen. Distractor was positioned parallel to the lower border of the mandible.

The test animals were not fed the day before the procedure. The subjects to be operated on were randomly selected without knowing which group they belonged to. For general anaesthesia, 50 mg/kg ketamine HCL and 8 mg/kg xylazine HCL were administered intramuscularly to all animals. Local anaesthesia with 0.5 ml of articaine containing 1:200000 epinephrine was applied to the surgical area. After shaving the left mandible of the rabbits and providing aseptic conditions with povidone iodine, a 3 cm long linear incision was made at the inferior border of the left mandible. A full-thickness flap was elevated. The osteotomy line was performed along the premolar tooth and the mental foramen. Before the osteotomy was performed, a custom-made titanium distractor that could be lengthened by 10 mm was positioned parallel to the lower border of the mandible with six titanium mini-screws (Figure 3). Then, the bone osteotomy was performed with the help of fissure burs and osteotomes under sterile saline irrigation and taking care to avoid mental nerve damage. The incision area was closed in layers with 4/0 polyglactin 910 sutures. After the latency period, the distraction protocol was applied for ten days with a distraction rate of 0.35 mm/12 h.

### Postoperative care

Tramadol 1 mg/kg and cefazolin sodium 50 mg/kg were administered intramuscularly twice a day for four days for postoperative pain and infection control. The animals were fed a soft diet for a week and their weights and nutritional status were checked daily by a veterinarian.

### ESWT application protocol after the osteotomy

In the O-ESWT1 and O-ESWT2 groups, ESWT with 500 impulses, 5 Hz, 0.19 mJ/mm^2^ energy flow density was applied to the distracted area on the 5th, 12th and 19th day after the osteotomy.

On the 43rd day after the osteotomy, all subjects were sacrificed using high-dose sodium pentobarbitone (Figure 1).

## Evaluation methods

### Radiological analysis

#### Computed tomography (CT)

**Figure 4.**
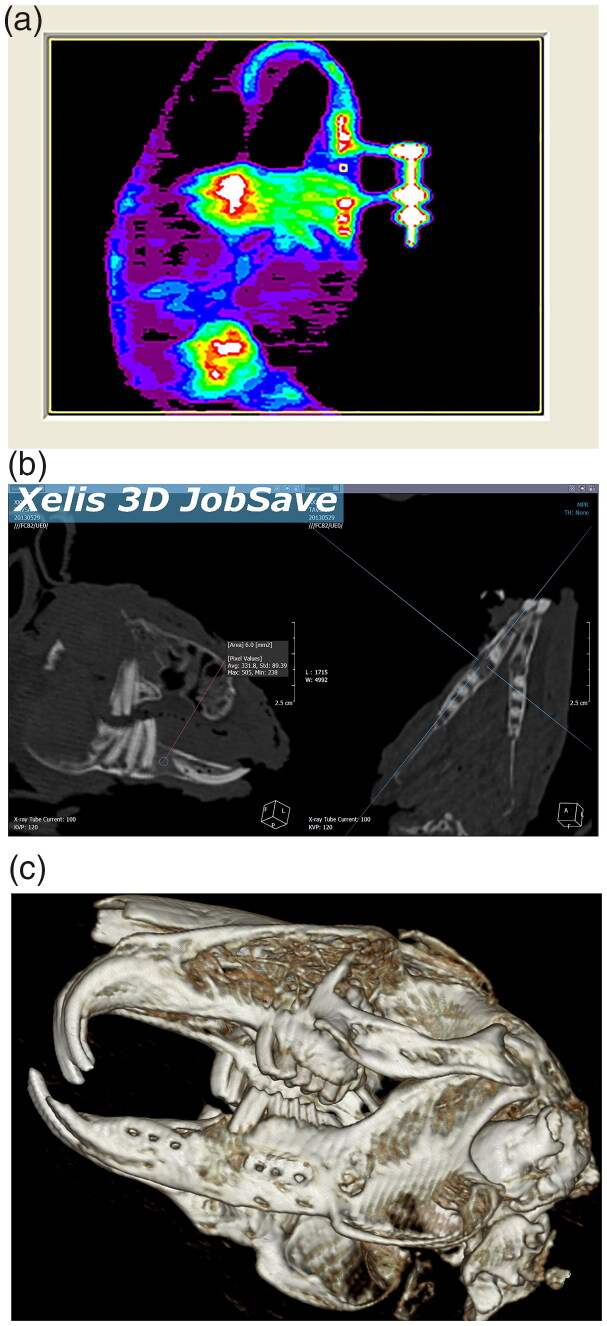
(A) DEXA. (B,C) Three-dimensional (CT) images showed healthly distracted bone and unilateral crossbite.

Computed tomography images were obtained from all the subjects for three-dimensional analysis of the distraction site immediately after sacrificing. High-resolution computed tomography (HR-CT) images of the mandibles were obtained using multi-detector computed tomography with consecutive 16-slice multi-detector spiral CT according to the screening procedure used for small animals (Figure 4(B)). Computed tomography analysis was taken to show the elongation in 3D as a result of distraction osteogenesis.

##### DEXA (dual-energy x-ray absorptiometry)

DEXA measurements were conducted double-blinded on the first week of consolidation (DEXA1) in high-resolution mode with the DEXA scanner, after intramuscular administration of 20 mg/kg of ketamine HCL and 5 mg/kg of xylazine HCL. The exact measurements were repeated (DEXA2) after sacrificing (4th week of consolidation). DEXA measurements were made at the center of the distracted area. The Bone Mineral Density (BMD) values were established using DEXA’s small subject animal setting (Figure 4(A)).

### Stereological analysis

A blinded histologist conducted the preparation and stereological examination of tissue samples. Soft tissues of the jaws (skin, muscle, fascia, mucosa and periostium) were removed and were fixed in formaldehyde (10%) for one week. Following fixation, samples were exposed to decalcification in formic acid (5%) for 21 d. After decalcification, the tissues and gradually dehydrated with alcohol and were embedded in fresh paraffin. Serial sections of 7-μm thickness were taken from each paraffin block. Paraffin blocks were sampled at a ratio of 1/10 according to the volumetric estimation procedure, and the first section was randomly selected. Sections were stained with hematoxylin-eosin (HE) and photographed with a color digital camera using a light microscope in a stereology analysis system.

Cavalieri method was applied to light microscope images for stereological evaluation of new bone tissue, connective tissue, and neoangiogenesis. Point-count test grids were used to establish the area in the sections. The following formula was applied to prove the point density, and an appropriate coefficient of error and coefficient of variation were established according to the pilot study [11]. This grid was randomly placed on the computer screen. The volume of the distracted area in all mandible sections was set with the following formula

Volume= t  ×  a/p  ×  ∑p

(‘*t*’, section thickness; ‘*a*/*p*’, area representing each point on the point counting scale; ‘∑*p*’, the total number of points corresponding to the distracted area).

### Statistical analysis

The data obtained from the densitometric and stereological evaluations were uploaded into the SPSS (Ver: 13,0, Illinois, USA) statistical program in a computer environment and compared with an ANOVA test. Pairwise comparisons between groups were made with the post hoc Tukey test. *p*-values less than .05 were considered statistically significant.

## Results

### Clinical observations and animal conditions

One subject in the O-ESWT1 group and one in the O-ESWT2 group died during the experiment due to infection and excessive weight loss. One rabbit in the Cont group was excluded from the investigation because the distractor came off. All other subjects tolerated the osteotomy and distraction protocol very well, and the distractor device remained stable until the end of the distraction.

### Radiological examination

In the CT images taken of the subjects, new bone formation in the distraction area had occurred ideally in all subjects. After DO, unilateral crossbite and excessive lengthening of the incisors were seen in all rabbits (Figure 4(B,C)).

In all subjects, bone mineral density (BMD) values were established with DEXA measurements made at the end of the 1st and 4th weeks of the consolidation period (Table 1). According to all measurements made at both the end of the 1st and the 4th weeks; the highest BMD value is seen in the Cont group, followed by the O-Cont, O-ESWT1 and O-ESWT2 groups. There was a statistically significant difference between the Cont/O-ESWT1 groups and the Cont/O-ESWT2 groups in terms of BMD observed on the 1st week, and between the Cont/O-ESWT2 groups and O-Cont/O-ESWT2 groups in terms of BMD observed on the 4th week (*p* < .05).

**Table 1.** BMD data obtained from DEXA examinations at 1st and 4th week of consolidation (g/cm^2^).

	Cont	O-Cont	O-ESWT1	O-ESWT2
First week	0.69 ± 0.09	0.56 ± 0.08	0.47 ± 0.11	0.46 ± 0.07
Fourth week	0.76 ± 0.04	0.69 ± 0.10	0.64 ± 0.04	0.53 ± 0.14

In the intragroup comparison, only in the O-ESWT1 group was a statistically significant difference between the BMD results on the 1st and 4th weeks of the consolidation period (*p* < 0.05).

### Stereological examination

**Figure 5.**
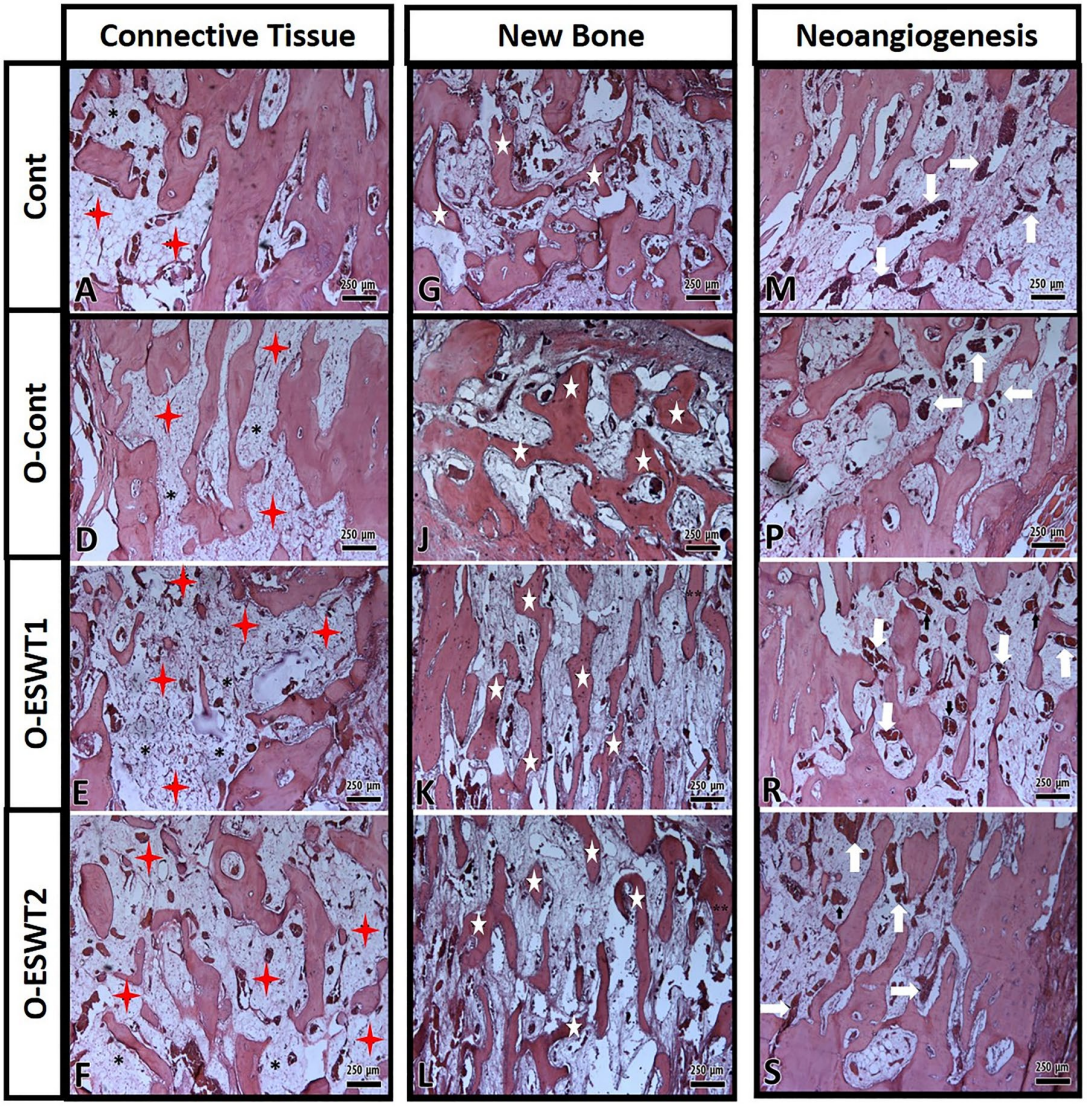
Histological image showing connective tissue areas of (A,D–F). The connective tissue areas are marked with red star. New bone areas have been shown in (G,J–L) images. New bone areas are marked with a white star. New vessel areas have been shown in (M,P,R,S) images. New vessel areas are marked with white (→). (original magnification ×5, hematoxylin-eosin).

Stereological examination showed new bone tissue, connective tissue and neoangiogenesis in subjects sacrificed after the distraction’s completion (Figure 5). All stereological results are stated in Table 2.

**Table 2.** Tissue volumes obtained from the stereological examination (mm^3^).

	Cont	O-Cont	O-ESWT1	O-ESWT2
New bone	0.20 ± 0.02	0.23 ± 0.03	0.31 ± 0.03	0.32 ± 0.03
Connective tissue	0.35 ± 0.02	0.41 ± 0.07	0.59 ± 0.10	0.51 ± 0.07
Neoangiogenesis	0.1 ± 0.02	0.08 ± 0.02	0.11 ± 0.01	0.09 ± 0.02

The highest new bone volume among groups was detected in O-ESWT2 and the lowest in Cont. Statistically significant differences were observed between O-Cont/O-ESWT1 groups and O-Cont/O-ESWT2 groups, Cont/O-ESWT2 groups and Cont/O-ESWT1 groups in terms of new bone tissue volume (*p* < .05).

The highest connective tissue volume among the groups was detected in the O-ESWT2 and the lowest in the Cont. The differences between Cont/O-ESWT1 groups and Cont/O-ESWT2 groups, O-Cont/O-ESWT1 groups were statistically significant (*p* < .05).

The highest neoangiogenesis among the groups was seen in O-ESWT1 and the lowest in O-Cont. A statistically significant difference was observed between the O-Cont/O-ESWT1 groups (*p* < .05).

## Discussion

The clinical success of DO is highly dependent on the condition of the bone microstructure. In situations such as osteoporosis, osteopenia and diabetes mellitus [12], which cause deterioration of bone microstructure, bone healing may cause some complications. Osteoporosis is a systemic disease in which the bone formation-resorption cycle and balance is disrupted in favor of destruction. In this disease, antiresorptive drugs (bisphosphonate-denosumab) and anabolic drugs (teriparatide) are used to prevent spontaneous bone loss [13]. However, the use of these drugs gradually decreasing because of insensitivity or tolerance and their serious side effects [14]. Biostimulation techniques such as ESWT, low-intensity ultrasound therapy, recombinant bone morphogenetic factors, low-level laser therapy, are methods is recommended to avoid complications in bone healing so that treatments can be successful [15–17]. Our study aim was to induce callus production and mineralization using ESWT in the osteoporotic experimental model, and as a result, accelerate maturation, improve its biomechanical properties, and shorten the consolidation process. Our study is the first to examine the effect of ESWT on the callus formation obtained in DO in osteoporotic subjects. The fact that all distractions occurred as planned and there were no cases of early ossification in any of the subjects in our study shows that the distraction protocol was successful. However, the lower bone density observed in the ovariectomized control group compared to the healthy control group indicates that the osteoporotic model was successfully implemented.

ESWT is routinely used in clinics for many musculoskeletal diseases such as plantar fasciitis, calcifying tendinitis, delayed unions and non-unions [17]. Moreover many research show that ESWT induces cell differentiation and neoangiogenesis in bone tissue increases the release of growth factors such as VEGF and BMP-2, and thus accelerates bone healing [18,19]. In addition, ESWT has been shown to have promising effects in osteoporosis, although studies are still very few. In a clinical study, it was reported that ESWT could improve markers of bone turnover in patients with low mineral density [4]. In a recent study, the therapeutic effect of ESWT on osteoporotic bone tissue in rabbits was investigated. It has been reported that ESWT applied to mesenchymal cells from the femur significantly increases the expression of osteogenesis markers such as ALP, Ocn, Osteoprotogerin and Runx2. In addition, it has been reported that osteoclast differentiation is inhibited by shock wave therapy. ESWT was applied to osteoporotic rabbits at an energy flow density of 0.12–0.5 mJ/mm^2^ every 3 d for 4 weeks, and it was reported that trabecular bone volume and mineral deposition increased significantly, and bone loss decreased [20]. In another *in vivo* study, it was observed that low-level shock waves (0.15 mJ/mm^2^) had a beneficial effect on the healing of osteoporotic fractures, leading to improved biomechanical properties, increased callus quantity and quality, and increased expression of bone-specific transcription factors [21]. In addition, it has been stated that it can accelerate callus maturation in the consolidation process in the DO model [15,22]. It is imperative to apply ESWT within proper parameters for this positive effect. Although necrotic changes occur at high energy flow density [15,20], applications with low energy flow density (regardless of the number of application sessions) have been shown to have an inducing effect on neoangiogenesis and bone regeneration [15,19,23]. In our study, we used ESWT with parameters that analyzed by us several times and whose positive results can be found in the literature [24]. In one of these studies, in which we examined the effect of ESWT on distraction osteogenesis, we determined that ESWT (twice 1000 impulses at 14 kV and 0.19 mJ/mm^2^ energy flux intensity) applied on the 1st and 4th days of the consolidation phase significantly increased bone mineral density and new bone tissue [19]. In another study, we found that ESWT (single dose of 1000 impulses 0.19 mJ/mm^2^ energy flux intensity) applied on the 1st day of the consolidation phase induced both new bone volume and new capillary formation [23]. In a different study conducted in the rat mandible, it was observed that a single dose of ESWT performed during the active distraction period significantly increased bone mineral density and new bone formation [25]. Guided by these studies, multiple sessions of ESWT application protocol were determined. Also ESWT parameters previously shown to be useful were preferred.

Studies have reported that shock waves cause an increase in cortical and cancellous bone volume, thus amplifying the mechanical properties of bone in areas that have not yet undergone surgery [26], inducing the proliferation of cambium cells in the periosteum, and increasing the periosteal thickness [27]. Studies report that the proliferation phase is the most significant period for bio-stimulatory methods [28]. For these reasons, ESWT was applied once a week for three weeks before and after the osteotomy in this study. With the ESWT application before the osteotomy, we aimed to increase the structure and mechanical properties of the bone with impaired microstructure. We also aimed to induce angiogenesis and proliferation during regeneration by continuing the ESWT after the osteotomy. A significantly higher rate of new bone tissue was observed in both ESWT groups compared to the O-Cont group. However, no significant difference was observed in the O-ESWT2 group compared to the O-ESWT1 group. These results show that shock waves induce callus formation, but post-osteotomy applications do not have an additional positive effect on pre-osteotomy applications. In addition, another study reported that ESWT with the same parameters, performed in 2 sessions on the 1st and 4th days of consolidation, did not significantly affect new bone formation [19]. In contrast, it has been reported that single-session applications at the beginning of the consolidation can have a significant positive effect [15,23,25]. In a study investigating ESWT in the treatment of delayed unions, it was observed that shock wave therapy applied as a single dose and 2 weeks later to fracture sites significantly increased endochondral ossification [17]. These results show that if ESWT is to be applied during the postoperative period, it is more efficient to use a single session at the beginning of the consolidation period. Although this study showed that the bio-stimulatory effect of shock waves was due to pre-osteotomy applications, in our previous study, in which we applied the same study protocol to healthy rabbits, pre-osteotomy ESWT application did not have a positive effect on new bone tissue [24].

Interestingly, neoangiogenesis was not induced simultaneously in the O-ESWT2 group, which was observed to cause callus. However, the highest neoangiogenesis was observed in the O-ESWT1 group and significantly differed from the O-Cont group. This result indicates that ESWT induces more neoangiogenesis only after osteotomy. In addition, the statistically significant difference between DEXA measurements performed on the 1st and 4th weeks in the O-ESWT1 group suggests that shock wave application after the osteotomy is also beneficial. To fully understand the cause of these effects, it is crucial to examine the subjects who underwent ESWT only after osteotomy in addition to these groups and to study the mediators and signalling mechanisms that are thought to be effective in osteoporotic bone healing and neoangiogenesis for more precise results.

DEXA is a reliable diagnostic method that is commonly used to estimate fracture risk by determining the bone mineral density in osteoporosis patients [15]. According to the results of the DEXA examination, lower BMD values were detected in the osteoporotic control group compared to the healthy control group, as expected. Yet interestingly, although higher new bone formation was observed in both ESWT groups, BMD values were observed to be lower in both the early (DEXA1) and late periods (DEXA2) compared to the O-Cont group. In our previous study in which we examined the effect of ESWT on distraction osteogenesis in healthy rabbits, we found lower BMD and bone mineral content values in the ESWT groups compared to the control groups [24]. One reason may be that immature bone is seen more frequently in the early period and the early radiological findings are insufficient due to the lack of mineralization of this bone [25]. Another reason is that ESWT may have increased osteoclastic activity. However, studies have shown that ESWT increases osteoblastic and osteoclastic gene markers, but the osteoclastic effect is short-term and temporary [29]. Therefore, the first reason stated appears to be more likely.

The strength of this study is the use of doses of ESWT previously proven to induce bone regeneration and testing it in an experimental model in which bone regeneration is actively ongoing. Although ESWT was applied in 3 different periods after osteotomy, it could not be determined in which period shock wave therapy had a promising effect. In addition, the lack of a study group that applied ESWT only before osteotomy can be considered as a weakness of this study. The most critical clinical significance of this study was to demonstrate whether the healing and strength of osteoporotic bone tissue could be induced by ESWT. It is critical that this effect has been studied especially in situations where bone healing and mineralization are important, such as in distraction osteogenesis.

## Conclusions

It is noteworthy that these positive results were obtained especially after osteotomy. Therefore, it may be helpful to study the application of ESWT with one session only after osteotomy in osteoporotic patients. In addition, it is necessary to establish the most appropriate ESWT parameters for the induction of new bone formation, bone mineralization and angiogenesis.

In maxillofacial surgery clinics, minor surgical applications are avoided as well as major surgical applications in osteoporotic patients. As a matter of fact, this study was carried out to increase the success of major surgical procedures such as distraction osteogenesis in osteoporotic patients. For this, ESWT, which has been shown to be an osteostimulative method, was used. In clinical practice, it is thought that ideal bone healing capacity can be achieved by applying ESWT to osteoporotic patients.

## Figures and Tables

**Table:** 

Authors	Conception and design of study	Acquisition of data: laboratory or clinical	Analysis and interpretation of the data	Drafting of article and/or critical revision	Final approval of manuscript
**Enes Özkan**		**+**	**+**	**+**	**+**
**Erman Şenel**	**+**	**+**	**+**		**+**
**Mehmet Cihan BEREKET**	**+**		**+**	**+**	**+**
**Mehmet Emin Önger**	**+**	**+**	**+**		

## Data Availability

The data that support the findings of this study are available from the corresponding author, [Dr.Enes Özkan], upon reasonable request.

## References

[1] NIH Consensus Development Panel on Osteoporosis Prevention, Diagnosis, and Therapy Osteoporosis prevention, diagnosis, and therapy. JAMA. 2001;285(6):785–795. 11176917 10.1001/jama.285.6.785

[2] Kanis JA. Assessment of fracture risk and its application to screening for postmenopausal osteoporosis: synopsis of a who report. WHO study group. Osteoporos Int. 1994;4(6):368–381. 7696835 10.1007/BF01622200

[3] Block MS, Christensen BJ. Porous bone ıncreases the risk of posterior mandibular ımplant failure. J Oral Maxillofac Surg. 2021;79(7):1459–1466. 33785292 10.1016/j.joms.2021.02.039

[4] Wölfl C, Schuster L, Höner B, et al. Influence of extracorporeal shock wave therapy (ESWT) on bone turnover markers in organisms with normal and low bone mineral density during fracture healing: a randomized clinical trial. GMS Interdiscip Plast Reconstr Surg DGPW. 2017;6:Doc17. 29308349 10.3205/iprs000119 PMC5738494

[5] Huang HM, Li XL, Tu SQ, et al. Effects of roughly focused extracorporeal shock waves therapy on the expressions of bone morphogenetic protein-2 and osteoprotegerin in osteoporotic fracture in rats. Chin Med J. 2016;129(21):2567–2575. 27779163 10.4103/0366-6999.192776 PMC5125335

[6] Ye B, Li Y, Zhu S, et al. Effects of ıntermittent low-dose parathyroid hormone treatment on rapid mandibular distraction osteogenesis in rabbits. J Oral Maxillofac Surg. 2017;75(8):1722–1731. 28500874 10.1016/j.joms.2017.04.010

[7] Xie MK, Hu CB, Zhou B, et al. Effect of gene transfection timing on TGF-β1 expression in rabbit mandibular distraction gap. Genet Mol Res. 2017;16(2). 10.4238/gmr16029330 28407179

[8] Simpson AH, Kenwright J. Fracture after distraction osteogenesis. J Bone Joint Surg Br. 2000;82(5):659–665. 10963161 10.1302/0301-620x.82b5.9945

[9] Simplicio CL, Purita J, Murrell W, et al. Extracorporeal shock wave therapy mechanisms in musculoskeletal regenerative medicine. J Clin Orthop Trauma. 2020;11(Suppl 3):S309–S18. 32523286 10.1016/j.jcot.2020.02.004 PMC7275282

[10] Zhang LE, Weng C, Zhao Z, et al. Extracorporeal shock wave therapy for chronic wounds: a systematic review and meta-analysis of randomized controlled trials. Wound Repair Regen. 2017;25(4):697–706. 28759136 10.1111/wrr.12566

[11] Sahin B, Emirzeoglu M, Uzun A, et al. Unbiased estimation of the liver volume by the cavalieri principle using magnetic resonance images. Eur J Radiol. 2003;47(2):164–170. 12880999 10.1016/s0720-048x(02)00152-3

[12] Ozkan E, Evmek B, Bereket M. Is the reiteration of extracorporeal shock wave therapy beneficial to enhance the bone integrity in type 1 diabetic rats? Ann Med Res. 2022;29(10):1.

[13] Yang TL, Shen H, Liu A, et al. A road map for understanding molecular and genetic determinants of osteoporosis. Nat Rev Endocrinol. 2020;16(2):91–103. 31792439 10.1038/s41574-019-0282-7 PMC6980376

[14] Compston JE, McClung MR, Leslie WD. Osteoporosis. Lancet. 2019;393(10169):364–376. 30696576 10.1016/S0140-6736(18)32112-3

[15] Lai JP, Wang FS, Hung CM, et al. Extracorporeal shock wave accelerates consolidation in distraction osteogenesis of the rat mandible. J Trauma. 2010;69(5):1252–1258. 20404761 10.1097/TA.0b013e3181cbc7ac

[16] Jiang X, Zhang Y, Fan X, et al. The effects of hypoxiainducible factor (HIF)-1α protein on bone regeneration during distraction osteogenesis: an animal study. Int J Oral Maxillofac Surg. 2016;45(2):267. 26508376 10.1016/j.ijom.2015.09.021

[17] Kobayashi M, Chijimatsu R, Yoshikawa H, et al. Extracorporeal shock wave therapy accelerates endochondral ossification and fracture healing in a rat femur delayed-union model. Biochem Biophys Res Commun. 2020;530(4):632–637. 32762942 10.1016/j.bbrc.2020.07.084

[18] Wang CJ, Wang FS, Yang KD. Biological effects of extracorporeal shockwave in bone healing: a study in rabbits. Arch Orthop Trauma Surg. 2008;128(8):879–884. 18560855 10.1007/s00402-008-0663-1

[19] Onger ME, Bereket C, Sener I, et al. Is it possible to change of the duration of consolidation period in the distraction osteogenesis with the repetition of extracorporeal shock waves? Med Oral Patol Oral Cir Bucal. 2017;22(2):e251-e257. 28160590 10.4317/medoral.21556 PMC5359710

[20] Li B, Wang R, Huang X, et al. Extracorporeal shock wave therapy promotes osteogenic differentiation in a rabbit osteoporosis model. Front Endocrinol. 2021;12:627718. 10.3389/fendo.2021.627718 PMC8027252 33841330

[21] Mackert GA, Schulte M, Hirche C, et al. Low-energy extracorporeal shockwave therapy (ESWT) improves metaphyseal fracture healing in an osteoporotic rat model. PLOS One. 2017;12(12):e0189356. 29232698 10.1371/journal.pone.0189356 PMC5726728

[22] Bulut O, Eroglu M, Ozturk H, et al. Extracorporeal shock wave treatment for defective nonunion of the radius: a rabbit model. J Orthop Surg. 2006;14(2):133–137. 10.1177/230949900601400205 16914775

[23] Bereket C, Çakir-Özkan N, Önger ME, et al. The effect of different doses of extracorporeal shock waves on experimental model mandibular distraction. J Craniofac Surg. 2018;29(6):1666–1670. 29742568 10.1097/SCS.0000000000004571

[24] Senel E, Ozkan E, Bereket MC, et al. The assessment of new bone formation induced by unfocused extracorporeal shock wave therapy applied on pre-surgical phase of distraction osteogenesis. Eur Oral Res. 2019;53(3):125–131. 31579893 10.26650/eor.20190041 PMC6761485

[25] Ginini JG, Emodi O, Sabo E, et al. Effects of timing of extracorporeal shock wave therapy on mandibular distraction osteogenesis: an experimental study in a rat model. J Oral Maxillofac Surg. 2019;77(3):629–638. 30121246 10.1016/j.joms.2018.07.018

[26] van der Jagt OP, Piscaer TM, Schaden W, et al. Unfocused extracorporeal shock waves induce anabolic effects in rat bone. J Bone Joint Surg Am. 2011;93(1):38–48. 21209267 10.2106/JBJS.I.01535

[27] Kearney CJ, Hsu HP, Spector M. The use of extracorporeal shock wave-stimulated periosteal cells for orthotopic bone generation. Tissue Eng Part A. 2012;18(13–14):1500–1508. 22519654 10.1089/ten.TEA.2011.0573

[28] Barnes K, Lanz O, Werre S, et al. Comparison of autogenous cancellous bone grafting and extracorporeal shock wave therapy on osteotomy healing in the tibial tuberosity advancement procedure in dogs. Vet Comp Orthop Traumatol. 2015;28:207–214. 25899991 10.3415/VCOT-14-10-0156

[29] Atsawasuwan P, Chen Y, Ganjawalla K, et al. Extracorporeal shockwave treatment impedes tooth movement in rats. Head Face Med. 2018;14(1):24. 30419912 10.1186/s13005-018-0181-5 PMC6233511

